# Valedictory Address Delivered to the Graduating Class of Rush Medical College

**Published:** 1866-02

**Authors:** J. Adams Allen

**Affiliations:** Prof. of Theory and Practice of Medicine, etc.


### CHICAGO MEDICAL JOURNAL.

Vol. XXIII. FEBRUARY, 1866. No. 2.

### ORIGINAL CONTRIBUTIONS.

*Valedictory Address delivered to the Graduating Class of Rush Medical College, at the Twenty-third Annual Commencement, held on the Evening of Jan. 24th,* 1866, *at Smith & Nixon's Hall.* By J. Adams Allen, M. D., LL. D., Prof. of Theory and Practice of Medicine, etc.

Gentlemen of the Graduating Class—In the absence of the distinguished President of the College, the courtesy of my colleagues has devolved upon me the pleasant duty—a duty sanctioned by time-honored custom—of welcoming you to the ranks of a noble profession, at the same moment we bid farewell to you in the capacity of students, for this day you doff the mantle of professional youth, to don the toga of professional manhood.

To one familiar with occasions like this, the thoughts which are necessarily awakened by its associations, will savor of the hackneyed and commonplace, and it is presumedly difficult to get above or below the level. It is difficult to say true things which are new, although it is surprisingly easy to say new things which are *not* true. But once more, before escaping completely from your present speaker, you must permit him to assume the *role* of Mentor, even though the parchments you have just received may be urged as a properly attested statute of limitations against the infliction.

You have just now been created "peers of the realm" of Rush

Medical College, after thorough readings of your several bills, and the Engrossing Clerk has carefully registered your names and seen them duly attested. We congratulate you on this promotion, and believe you will do honor to your election. Meanwhile, moderate your natural feelings of exultation at having thus successfully traversed the rough and rugged road of your pupilage to this brief resting place, by the reflection that, with your new honors and privileges, have come also new and inexorable demands for future effort. In these days nothing is respected, save for a fleeting moment, but that which proves itself respectable. "The dignity of our glorious profession" is a mouth-filling phrase which always elicits applause and hand-clapping at medical societies and conventions, and some men get notorious, if not famous, by rolling its syllables (as other men do sin) like sweet morsels, over and under their tongues. But this is a wicked world, "far gone from its original (Æsculapian) righteousness," and does not believe in glory of any sort, save as a political or otherwise marketable commodity.

By proper arrangement of affairs you can make your diploma a little deed to inestimable wealth; by improper consideration thereof, you would cheat whoever you might wish to sell it to, even though you got nothing but an Esau's mess of pottage. Your parchments are the challenge to all mankind that you have laid aside your original besetting sins of ignorance and prejudice, that you have practiced your muscles, your nerves, and especially your eyes and ears, and are now ready to quit yourselves like athletæ in the game of life. What you will *do* remains to be seen.

Your *Alma Mater* presents each of you with these parchment shields, and, like her of olden times, commands you to return from the battle of life with, or upon them—with them inscribed with noble histories of new triumphs over disease, and especially ignorance, the worst of all maladies. For whatever may be the common prejudice and notion with regard to the true physician's business; whatever building of Babel towers of nosology, each brick a squared and jointed disease; whatever concoctions of mysterious mixtures "warranted to cure" (or kill, as the case happens;) whatever gibes and sneers, because men will die in spite of our glorious profession, the truth remains, that the true physician's business is to combat the ignorance, both involuntary and willful, which everywhere abounds. Be assured this generation will call upon you for a "sign" of your mission, else they will not believe you one of the prophets; neither will they give you profits in anywise. Woe unto you if you have no sign save yonder roll you grasp so contentedly.

Men have a peculiar faculty of permitting in themselves a weakness and littleness and meanness and ignorance which they quickly spy out and loudly reprobate in others. Though, as Carlyle says, quack and dupe are upper and under sides of the same coin, yet the reversed faces, by a curious moral strabismus concentrate a telling and judging glance upon any other counterfeit. He is simply a great fool, who in these days undertakes to dupe a very foolish public. The foolish public has a very foolish trick of being honest—to its own interests when it knows them. There is no use in wasting either lamentations or profanatics, as the manner of some is, over this popular folly of to-day, which will be chased to death and torn to pieces by the bigger popular folly of to-morrow. You may attack it by preambles and long-winded resolutions in solemn conventions; you may wax lugubrious over it in very *b*athetic orations; you may lash it with invectives and frightful adjectives in state papers or newspapers—but it will not "down." Some then have thought to tie it up in strings of red tape by legislative enactment, that no ill-mannered and worse tempered dogs shall bark at the heels of our Sir Oracles, and especially that these curs should not make free with the Æsculapian dinner. But Tray, Blanche and sweetheart—curs of high and low degree—from Indian to infinitesimal, still remain dear to the popular heart, and poodles and pups of all shades and configurations, are yet carried in hysterical arms, and fastened to the offal carts of medical scavengers.

Briefly—Physicians should be men, oaken and iron men, capable of being and doing, whatever fever thrills the public pulse, or enervates the public brain. Hot-house plants are very pretty in their way, but scarcely the thing for outdoor exposure. Alas for the fantasy that would throw around a stalwart graduate of old Rush Medical College the brittle protection of legislative statutes, or bind up his strong limbs with the flimsy cords of Trades Union Societies. The most of you are now thinking of "openings for business," some place where you can carry out practically what you believe you have received scientifically. So far as this is concerned, one at least of your old professors advises you in this wise: Do as the petroleum men do—take the surface indications and then bore. Old Dr. Sam. Johnson said, "if you have got anything to do, sit down doggedly and do it." Don't whine, but work at it. This man will disappoint you, and the other man will fail you; don't depend upon anybody but the present graduate of Rush Medical College in good standing. "The rolling stone gathers no moss." There is no opening for either of you; you have got to erect your derrick, and *bore* for medical success. To look for an opening, and wait for an opening, is like him who waited for the water to run by, that he might pass over the river dry-shod. The surgeon in the last war, who broke up his old connections to go thither, and now that it is over, of course comes to Chicago as the place wherein to win new triumphs over fortune, success and disease, well understands this in the abstract. Several hundreds of them are, and have been trying it. The river will run by ultimately. God help him who waits. "Openings" are only to be found as prizes in a lottery; they are to made as one digs a well. It is *well* if you make it. Find the place that suits you, and then erect the Æsculapian derrick.

Business of whatever kind requires "pluck"—requires "nerve." It is all well enough to talk of "modest merit," and of the grand fact that "truth is mighty, and will prevail," but in these desperate times it is gospel truth that a candle set under a bushel is a solecism, an "anti-new dispensation," and an epitome of darkness. Practically, you must put your candle, rush-light, farthing or Drummond, upon a night stick, that it may be seen of all men, and especially of all women.

Somebody wrote a novel—which I have never read—entitled, "What will he do with it?" The answer to my mind is as clear as sun-light. He should do totally different from him who wrapped his talent in a napkin and hid it. He must nourish it and cuddle it. He must increase it by all the powers which he hath or can obtain. Otherwise, he profiteth not, and is in danger of the outer darkness and bonds.

Success, my beloved, is not a thing of chance. Chances make the fool's paradise. The part of wisdom is to convert chances into certainties, and make fortune the slave of the wheel, and not its mistress.

Success is a word which men interpret differently, and perhaps it were well that we canvassed its meaning before passing on to higher themes. One man counts success the accumulation of dollars and mortgages and bonds. Another counts it secured when all men applaud the effort and sustain the claim for position and fame. But your present speaker has a different idea of this from either of these. With deepest reverence he turns to Holy Writ, and remembers that, in the mysterious designs of Providence, man was permitted to fall from Paradise even to secure something beyond happiness and material felicity,—something beyond unearned satisfaction of material wants—something beyond the exemption from pain and death. The grand allegory teaches that neither brow-sweat nor brain-sweat, neither pain nor death were penalty enough to dissuade man from securing the high felicity of knowledge. The primeval curse has become a perennial blessing.

"Growth is the attribute of all the mind's attributes." You may confine the body in poverty, in pain, in prisons, but you cannot control the human spirit. You may pile creeds and usages, forms and dogmas, and erect your columns of Hercules—may even place Heaven's angels with blazing swords everywhere opposing, yet the human mind shall transcend them all, and out from the impossible of our age shall the creative intellect build the possible, the every-day, the common-place of the next.

With God all things are possible, and as He has set no limit to the development of the human intellect, so, with profoundest reverence be it spoken, it becomes possible for him into whose nostrils He breathed the breath which brought to being a human soul, to overcome the apparently inevitable, and cause the dream of philosophy to welcome the work of the day laborer.

Success is the accomplishment of the highest truths which Revelation and knowledge teach you. Between these there is no incompatibility. The truths which Moses brought down from Sinai, and David celebrated in immortal psalm—which Christ promulgated from Palestine, and Paul sustained with unequaled and inimitable writings while martyrdom stood by his side—are none of them outside the sphere, or opposed, in the slightest degree, to the studies which I urge upon you. "I am a man," said the old Roman writer, "and nothing pertaining to humanity do I consider foreign to me." It is a foul aspiration, that we cast back with unmingled scorn, which claim that medical men are skeptical or prone to so-called materialism. None like them appreciate man and nature.

"This most excellent canopy, the air, this brave o'erhanging firmament, this majestic roof fitted with golden fire! What a piece of work is man! How noble in reason! how infinite in faculties—in form and moving how express and admirable—in action how like an angel—in apprehension how like a god—the beauty of the world, the paragon of animals!"

We delve deep in the mysterious organic structure, but the gross scalpel throws back from its polished surface the reflection —"There is a God who made all this." The unaided vision and the microscope alike bring up to the intelligent sense but one report: "The material form is but the visual expression of the grand artistic thought." The created form mirrors the Creator, and the glitter of new discovery is whelmed in the ocean of knowledge upon the sands of whose shores our footsteps leave but a passing imprint.

Primarily, the physician should know exactly how the human body is organized; he should understand Anatomy thoroughly. Though he speak with the tongue of angels, and understandeth not Anatomy, he is nothing. Next, he should know the science of the organs in action, or Physiology. Anatomy is of dead things—Physiology of the living. The physician who does not understand both of these—I do not care whether he has secured one or half a hundred of yonder parchments—he has no right to meddle with healths or lives. Pile diplomas and popular reputations, heaven high upon him, and they only crush him deep beneath decent contempt.

Next he should understand Chemistry in all its range and minutiæ, the laws of all forces or imponderable agencies with the actions and reactions of all elements upon each other, and particularly upon the human body, thus including what we call *Materia Medica*, and rightly also *Medica Alimentora*, or Food. What right have you to meddle with forces or matter, unless you understand them? I will excuse here a liberal skepticism of old Witts' fables about sundry things, good for divers diseases, but I will not excuse, nor in any wise commend herein, dabbling with what you fancy remedies for diseases of which you know nothing, in bodies of which you know less.

With these things you must combine those mechanical excellences requisite in surgery, and that other fine and ("nameless forever here") each of which requires perfect acquaintance with the science before mentioned, together with the skill of the artist and the calm serenity of the mythic Fates.

The long list of so-called specialities are but parts and parcels of these. "Let each be fully persuaded in his own mind" that he has imbibed nutriment from the trunk and branches of the great tree of medical science, before he undertakes to flourish as one of its blossoms.

But the medical man, thoroughly as he may understand the curriculum of studies ordinarily traversed in medical colleges, by no means is yet prepared even for professional success. It is trite to the last degree of commonplace to say that you have really but just commenced your professional studies. We have scarcely more than suggested the elements and pointed out to you the paths of study. We have opened to you the doors of the Æsculapian temple, and given you a clue to its halls and apartments. It is not so labyrinthine as the structure which lodges the Minotaur of the old fable, nevertheless it will tax all your faculties to explore. We have given you some keys and charts. God speed you on!

I commend you, further, ample reading of the best works upon the Philosophy of the Human Mind. The fault of metaphysicians or psychologists is, that they are not physicians or physiologists. The fault of physiologists is that they are not psychologists. Man is a wonderful admixture of matter and force, and he who would control either his body or spirit should understand the laws of both. Professionally I commend this idea, not only to you graduates, but to statesmen and divines. Miraculous, almost, seems he potency of the human spirit upon the body which it wears, and great is the sway of the form over the force which inhabits its atoms. The great law that "action and reaction are equal" nowhere finds more admirable illustration. The black blood of dyspepsia and glandular inaction infects the mind with melancholy, fantasies and disordered notions, which neither God nor men admire; whilst on the other hand the subtle spirit of life can control, either to its destruction or to its repair and health, the crude elements which build up the human frame. The diseases of the world, without any recurrence to a transcendental philosophy, are a reflex of the mental habitudes, and the great many influences which shape the character of the different ages of the world. The reciprocal influences of mind and matter we study as scholars with regard to the individuals, but are exceedingly liable to overlook in relation to the masses of mankind.

Yet it is safe to say, that these largely control the phases of disease, and must modify both diagnosis and treatment. It becomes, therefore, your duty to study these influences and ponder upon their results. Some one truly said: "The individual man is an enigma, the mass is a mathematical problem." Then only will there come an exact and generous medical science, when this truth is recognized—when the microscopic littleness of old diagnoses there shall be superadded those grand, general and comprehensive views which shall grasp in a Newtonian system the great productive agencies of diseases. Medical science stands upon the verge of glorious discoveries. Little by little it has been gathering the scattered links, and laboriously working out the subordinate problems. It has been passing through the fire of ridicule, and too often worn longer than tolerably bearable the Nessus' shirt of self-conceit. But more than a twilight is now breaking through the darkness and mists of the ages of long ago. As the astronomers have mapped out the apparently trackless heavens, and measured the stars in their courses—as geographers have counted the acres of the continent and islands, and have made charts of the currents of the supposed incomprehensible oceans,—so shall we, ere long, pluck from diseases the heart of their mystery, and establish their laws, side by side in certainty, with those whose secrets have already been wrested from great Nature. But in order to accomplish this, we must continually remember that there is a mind as well as a body, and that it is but a half truth—almost worse than a falsehood—which divorces the two in study. I therefore recommend you to study men as men—to remember the patient in the subject, the healthy in the sick. Observe all influences, political, civil, moral, domestic, personal, which operate upon him. To do this advantageously, or with even approximate correctness, you must not only be intelligent, but "men of affairs"—thoroughly posted in up the literature and science of the times. You will thereby gain personal culture as well as professional advantage.

I even insist upon your reading the daily newspaper, not for its probably vicious political views, but because it is a chart and chronicle of the times. In the older days, knowledge was mainly confined to books, and the antique bibliomaniacs even now have a fashion of deriding the newspaper; but it is safe to say that at least a score of newspapers may easily be selected, in this country alone, which contain on the average better writing, rhetorically, and certainly vastly more valuable information in a single issue than can be found in most of the so-called classical volumes, which would, if now published for the first time, perish upon the booksellers' shelves.

A similar remark may be made with reference to many or most medical books as compared to the current journals. The books smell of the mouldy centuries—often effete or worse, but some of the journals—perhaps *rari nantes in gurgite avsto*—are alive with the spirit of the age. They have a vitality, a freshness and progressiveness about them, which should commend them both to your subscription and regular perusal. They will keep alive the fires now animating your professional youth, and prevent you from lapsing, as is, alas, too often the case, into a slumberous beatitude of professional hybernation. Keep your wits about you, men; keep your eyes and ears open. Read daily and observe constantly; and don't say you have no time, the excuse of sluggards, tobacco smokers, billiard players, bar-room politicians, wayside gossips, and dog and gun fanciers. You have no time to do otherwise. The physician, of all men, must be clear-headed, well informed, capable of taking any position in society, or even in politics, but invariably declining the latter. Alas, how many good men I have seen spoiled by neglect of this last suggestion. Keep out of it as you value your profession, as you prize a good name.

There has somehow got into being the idea that the study of our profession is a hardening process; that in familiarity with its details and experiences is involved a loss of the finer feelings and delicate sentiments which ennoble and refine human nature. The scalpel and the miscrocope which disclose, whilst they do not explain the material mechanism, are believed to determine a callousness of perception which prevents admiration of its exquisite structure; and beyond this, that those experiences which bring daily, and almost hourly, before us the sad evidences of the frailty and ignorance of the race tend to provoke a contempt for human nature which represses the growth of the higher emotions which distinguish the humanitarian and philanthropist. And indeed, when we recognize the great fact that individual violations of the great and well known laws of nature, and that the pestilences and epidemics, at the very mention of which the popular pulse throbs heavily, and the hearts of all men fail them for fear, though sometimes springing from obscure causes, are nourished to ten fold, nay, a thousand fold more destructive energy by their own wilful and stubborn negligence and ignorance—there is a liability for the professional mind to turn with disgust from the seemingly hopeless task of detaching Ephraim from his idols. Let the cloud now hanging threateningly upon the Eastern horizon, and whose lurid shadows just begin to fall upon the people of this continent, fail to launch its thunderbolts upon us in the coming spring or summer time, and the municipal dogs will return to their political vomit, and physicians be, as of old time, despised as croaking Cassandras. Why, this great city of Chicago, a little while since, in an ecstacy of fright over the threatened advent of cholera, and ready to do almost anything to prevent, as when

> "The devil was sick, the devil a monk would be;
> The devil got well, the devil a monk was he;"

has had in a few days of cold weather its gigantic twenty-five thousand dollar energies (that is about twelve and a half cents a head for its population) crystallized into some half a dozen dilapidated swill and ash wagons, drawn by half blind and wholly spavined horses. Meanwhile, the collections thereof are permitted to be dumped into some Healy Slough, or similar receptacle of all nastiness, to send out concentrated poisons with the first vernal showers and sun.

But so far from the profession being rendered callous and indifferent by these things, we assert that they only develope and cultivate in them a righteous wrath which the sun does not go down upon. And so all along the track of human ills, albeit the offspring of man's own wilful ignorance, obstinacy or stupidity, true medical philosophy scatters rays of benevolent light. Although tempted at times to adopt the theory of the "Vestiges of Creation," that men have progressively been developed from "monads and reptiles and chimpanzees, to their present form," and that some have had "arrest of development" on their passage through the typical forms, we still adhere to the higher doctrine that man was created as he should be; that for some great purpose he was permitted to fall from his high estate, and is now slowly, but surely clambering up again. It is somewhat difficult, we admit, always to entertain a generous confidence in the latter statement, but we nevertheless accept it as an inexorable fact in our philosophy and are comforted thereby. A man in any business or calling who neglects the ordinary elements of culture must expect to pay the penalty in stunted mental growth. The medical man who sinks his justly entitled liberal profession into the mere trade whereby he lives, and the art whereby he accumulates houses and lands, and Government bonds, will of course, become hard and cold, illiberal in heart and mind. But this is clearly not the fault of the art or science.

Bunyan depicts the old man with his muck rake, industriously forgetting to look up at the angels who holds out the celestial crown just above his head, and within his reach if he would look up for it. And we have thought now and then, that some medical men have in like manner lost glorious opportunities for enlargement of perception, refinement of sense and the crown of large and liberal reason, by forgetting the existence of other knowledge and arts whilst raking among the debris of forgotten lore, or burrowing, molelike, away from the sunlight of modern thought among the detritees and fossils of the effete ages.

The first requisite to a medical gentleman is that he should be capable of thinking—the next, that he should know how to think, and then that he thinks of things worth thinking of. The world knows but little of anatomy, but there are not wanting men of shrewd sense who know how to recognize *brains*. You may catch the ears of the groundlings and even the patronage, temporarily, of the mass by the assumption of a hypothetical professional wisdom hidden in sesquipedalian phrase and words of learned length and thundering sound—particularly if you have opportunity to air them before courts and juries, but there will not be wanting divers plain men who will detect the shallow imposture and pluck off all mere jackdaw feathers. No man, as Bacon said, can truly understand science until he rises to the level of all sciences, and strikingly is this true of the medical man. His faults of primary education must be corrected by mature observation and study; his professional acquirements must of course be accurate and extensive; but beyond this, he must familiarize himself with *belles lettres* and the records of thought and action.

Cultivate personal character and surround your every day life with the atmosphere of literature and high art. I commend even poetry and music and the charming relaxation of social concourse with the intelligent and accomplished in any of the higher spheres of life. The physician in a sick room should be a gentleman; that is the soul of the code of ethics. Out of the sick room he must be nothing less. The cold-blooded boor has no business to be a physician, though he has anatomy and all the medical "ologies" at his tongue and finger's end. He only plays on the "harp of a thousand strings" as though it were a threshing machine or a Chinese gong. I pray you avoid this, in daylight and darkness. On the other hand, remember that no true gentleman ever suffers himself to degenerate into a dandy or a dancing master, or puts on owlish airs of a dignity to which he is not entitled. What sometimes passes for dignity is pretty often stupidity in a transparent mask.

Gentlemen, the session of Rush Medical College which this day closes, has been, as you are aware, unmarked by any prominent incident; neither severe sickness nor death has been among you and harmony has everywhere prevailed. The course of instruction from the first lecture to the closing, has moved on as steadily and regularly as the course of a river to the sea, with broad and deep channels and nothing to oppose. Your zeal and assiduity, your constant courtesy and attention to the lectures, and finally your evident mastery of the topics upon which you have severally been examined, give the Faculty and Trustees peculiar pleasure in awarding to you the honors in their power to bestow. From many of you we shall expect to hear high things—from all of you, good things. As we have no doubt of your ability, we cherish no misgivings as to your success. You will gain respect for yourselves, and your *alma mater* will share each laurel.

We miss from our own number one familiar face, and you participate with us in the regret that even his powerful frame is not wholly proof against disease. For nearly a quarter of a century has Prof. Brainard not only occupied, but *filled*, the position of a professor of Surgery in this College. How he has filled it, not only the alumni of this College, but all educated surgeons, not only of this country but of all Christendom, can bear testimony. Originally endowed with a masterly intellect, quick perceptions, great industry and an iron energy of will and purpose, he has already placed his name high upon the roll of those names which the profession "will not willingly let die."

His rare faculties of invention and genius in adaptation have added largely to the resources of surgery everywhere, whilst his unequalled powers as a teacher in his department, have done really more for the preservation of life and limb during the recent war alone, than would suffice to make a dozen reputations for the gazette or official bulletin. It is not claiming too much for him to say that he is not outranked by any living surgeon.

We cheerfully yield him the few months demanded for the restoration of his impaired health, and we look forward with great pleasure to the time when he will return from his European tour, prepared, not only to resume his position in the college, but again to give the profession the benefit of his large attainments and solid counsel. His absence must excuse these few words of eulogium, from one who knows well whereof he speaks, and that the daintiest sense of propriety cannot be offended by the tribute.

Gentlemen—The months of our association have gone rapidly into the past, and the moments of this, our last formal meeting, are slipping away from us, but we are assured that you will look back upon them with every renewing pleasure, even though gladly you go forth to the duties of a more active life. We commit to you the making of your own reputations, the honor of the profession, and beyond all, the care of lives and healths. Duties and responsibilities are not to be put on or off at your pleasure, like hats and coats, but must be worn forever. We send you to the fore front of the conflict with disease and ignorance. We know you will not skulk when cholera or other pestilence comes; and we believe that in the more constant and sterner battle with ignorance you will prove no cowards. For these life struggles we bid you *Farewell.*

